# Experiences of Second-Grade Primary School Children and Their Teachers in a Mind–Body Activity Program: A Descriptive Qualitative Study

**DOI:** 10.3390/healthcare12202095

**Published:** 2024-10-21

**Authors:** Yaiza Lopez-Sierra, Sara Trapero-Asenjo, Isabel Rodríguez-Costa, Gonzalo Granero-Heredia, Yolanda Pérez-Martin, Susana Nunez-Nagy

**Affiliations:** 1 Amavir El Balconcillo Nursing Home, 19004 Guadalajara, Spain; 2 Department of Nursing and Physiotherapy, Faculty of Medicine and Health Sciences, University of Alcalá, 28805 Alcalá de Henares, Spainsusana.nunez@uah.es (S.N.-N.); 3 Humanization in the Intervention of Physiotherapy for the Integral Attention to the People Group (HIPATIA) Group, University of Alcalá, 28805 Alcalá de Henares, Spain; 4 International Doctoral School, University of Almería, 04120 Almería, Spain

**Keywords:** mind–body therapies, mindfulness, emotional regulation, yoga, respiration, child, physiotherapy, qualitative research

## Abstract

Objectives: This study explored the experiences of second-grade children and their teachers who participated in a mind–body program to understand its impact on their development. Methods: A qualitative descriptive study was conducted. Student data were collected through seven focus groups, and semi-structured interviews were conducted with tutors in December 2021. Data analysis was performed following COREQ guidelines. Results: Three themes were generated: (1) competitiveness and restlessness in children: something needs to be done; (2) seeds sown and fruits harvested; and (3) it is necessary to advance from the occasional to the structural. Participants reported positive changes in areas of their development such as self-regulation, relaxation, attention capacity, and stress reduction. Improvements in interpersonal relationships and social skills were also observed. Conclusions: These improvements in emotional well-being and social skills highlight the importance of this type of intervention in the school environment.

## 1. Introduction

From birth to old age, a person experiences a constant process of physical, emotional, social, cognitive, and spiritual growth [1]. Human development across the lifespan is a fascinating process marked by a succession of stages, each characterized by its own set of challenges, achievements, and transformations [2]. The development of children in full involves the maturation of psychomotor, socioemotional, and cognitive areas according to the fundamental needs corresponding to each age [3].

The immediate environment of children between 6 and 7 years of age has perceived, in recent years, an increase in the difficulty they have in maintaining attention and concentration, to perceive their body and their emotions, as well as to express and regulate them latter [4]. Likewise, in recent years, an increase in disorders such as Attention Deficit Hyperactivity Disorder (ADHD) [5] has been described, which affects about 11.4% of children worldwide and is associated with a higher risk of suffering alterations in other areas of development and a decrease in quality of life [6]. This disorder harms children’s school and social development [7], and ADHD is associated with lower educational achievement [8].

The age of 6 to 7 years is a stage of many changes in the child’s environment; they develop their abilities, character, and autonomy more intensely [9]. According to the Ministry of Education, Vocational Training and Sports, six years of age is when compulsory education begins in Spain, and therefore, the demands of responsibility, planning, and control capacity increase. Thus, in the psychomotor area, the early years are vital for developing postural control [10] and gross motor skills such as jumping and running [3]. Progressively, these skills are refined, leading to more complex skills [11]. Failure to develop these skills could delay their progression [12] or even have repercussions in other areas [11]. In the emotional area, the importance of attachment figures [13], which facilitate the development of emotional regulation and self-control, understanding of social relationships and responsibility [14], and moral development [15], stands out. The cognitive area, closely related to the motor area [3], orchestrates actions or mental processes of acquiring knowledge and understanding through thought, experience, and the senses [16]. Cognition comprises the executive functions (involving perception, memory, and action [17]) that enable self-control through inhibition, planning, attention, and working memory [18].

The focus is on mind–body approaches to address the difficulties perceived in recent years [19,20]. These are defined as a group of strategies based on the principle that the mind and body are interconnected and can improve the physical and psychological well-being of human beings [21]. They take advantage of the benefits derived from the synergy between the body and the mind (the mind influences physical functions and vice versa). These approaches include mindfulness, yoga, conscious breathing, and guided imagery [22]. Mind–body therapies have been shown to lower inflammation markers and impact virus-specific immune responses following vaccination, although there is limited evidence indicating their effects on resting antiviral responses or quantitative immune measures [23]. Recent studies have shown that mind–body approaches have beneficial effects on young populations, including those suffering from ADHD [24]. These benefits are attributed to their positive effects on psychosocial, emotional, and neurobiological functioning particularly relevant in addressing the challenges posed by ADHD [24].

Mindfulness is the ability to deliberately bring attention to the experience of the present moment with acceptance, with curiosity, and without judgment [25] and has shown improvements in physical and mental health [26]. Yoga, for instance, is a physical practice involving the relationship between body and mind [19] that procures benefits in both areas [27]. Building on these principles, mindful breathing has benefits in academics [28] and at the emotional and physiological levels [29]. Central to many of these practices is body awareness, which involves the attentional focus and awareness of internal body sensations [30] and is a crucial element for self-regulation [31]. Complementing these approaches, guided imagery in children is particularly effective and has cognitive benefits [32].

Emotional affectations can cause physical symptoms, such as muscle tension and altered postures [33]. For more than 15 years, the role of physiotherapy in caring for children’s needs has been highlighted, with particular emphasis on mental health due to the close relationship between the body and the mind [34].

After reviewing the available literature, mindfulness and mind–body interaction techniques are found to have been studied more extensively in adults. Research abounds on mindfulness techniques, using breathing, body awareness, or yoga [35], and their benefits in different areas of children’s development [36,37], especially supporting full development as learners. However, the perceptions of children themselves and their teachers from a qualitative point of view are not yet known in depth.

The existing literature highlights a significant gap in understanding how mind–body approaches specifically impact children aged 6 to 7, a critical developmental stage characterized by increased demands in attention, emotional regulation, and social skills. Although numerous studies have explored mindfulness and related techniques in adult populations, there is a lack of qualitative research examining the perceptions of children and their teachers regarding these interventions. This age group is pivotal, as it marks the onset of formal education, where children’s cognitive abilities, emotional character, and personal autonomy are rapidly developing.

Gaining insights from the perspectives of both children and educators could reveal valuable information on the effectiveness and real-world applicability of mind–body strategies in educational settings. This qualitative approach provides a more nuanced understanding that quantitative outcomes alone cannot capture, offering insights into the lived experiences of participants that previous research has often overlooked.

The present study aimed to address this research gap by exploring the perceptions of 6- to 7-year-old children and their teachers who participated in a program led by the school’s physical therapist that focused on mindfulness, body awareness, conscious breathing, and emotional management. These insights may inform the development of tailored interventions to support better the holistic development of young learners facing contemporary challenges, potentially revolutionizing the approach to early childhood education and well-being.

## 2. Materials and Methods

### 2.1. Design

A descriptive qualitative study was conducted. This type of study uses a naturalistic perspective to understand the object of study in its natural context. It seeks to know the phenomenon through the experiences, events, and interactions in the phenomenon from the point of view of the participants [38]. It allows for a direct description of the phenomenon or a description of the interventions in order to improve them [39]. The consolidated criteria for qualitative research reports (COREQ Scale) were followed when writing the report [40].

### 2.2. Participants and Environment

The participants were all the students in the two classes of the second year of primary education (51 children between 6 and 7 years of age: 27 boys and 24 girls) (Table 1) and the two teachers. The program was developed at a Colegio de Educación Infantil y Primaria (CEIP) in Alcalá de Henares, Madrid, Spain. A CEIP is equivalent to a combined elementary and middle school in the United States, typically serving students ages 3 to 12. This particular CEIP is an inclusive public school that mainstreams students with various disabilities, including those children with physical, language, and cognitive challenges, alongside typically developing peers. The school is characterized by its commitment to the holistic development of its students, embracing an approach that respects diversity. It emphasizes the importance of academic content acquisition, promotes healthy habits, fosters social values, and prioritizes emotional management skills. The teacher of one of the classes had more than 30 years of teaching experience, specifically 14 years with this age group. The teacher of the other class had more than 30 years of experience, 10 years with this age group. Participants were recruited through non-probability and purposive sampling. The inclusion criteria were as follows: participation was voluntary, and participants (guardians) or their legal representatives (parents or legal guardians of the children) had to read the information sheet and sign the informed consent; participants had to have had some relationship with the program of mindfulness, body awareness, conscious breathing, and emotional management. The center’s physical therapist, who led the program in recruiting participants, was contacted. She had over 30 years of experience as a physical therapist in this educational center and more than 20 years leading workshops on mind–body relationship techniques (more than 7 with children). The physical therapist facilitated contact with the center’s management and the class teachers to manage the families’ permission for the interviews. The Territorial Center for Innovation and Training of Madrid positively evaluated the intervention program applied.

### 2.3. Program Description

The program consisted of 8 sessions that were about 45 min in duration. In each of them, the physical therapist carried out different activities in order to develop different objectives.

The program rules were established in the first session, and a climate of trust was created in the classrooms to foster group cohesion. Additionally, the children were introduced to mindfulness practices to promote emotion identification. It was essential to provide an introduction adapted to their level of understanding, while also emphasizing the importance of group cohesion as a key benefit of the activities [41].

In session 2, the practice of breathing was initiated to promote mindful breathing and work on attention and concentration through an exercise with colored stones. The children were also initiated in body scanning techniques to help them identify and become more aware of their bodies. This session focused on mindful breathing, which aims to provide physiological benefits by positively influencing the parasympathetic nervous system [27].

In session 3, they were taught the cardiac coherence technique adapted to their age to learn how to manage stress. They were also introduced to hakini mudra, a hand gesture used in yoga and meditation to enhance concentration and focus. It is combined with breathing, and this mudra facilitates cooperation between the two brain hemispheres, thereby helping to enhance children’s concentration and memory [42].

In session 4, the techniques of the previous days were reviewed, and a mindful movement exercise was performed. This exercise consisted of walking with mindfulness to continue practicing body exploration and promoting movement and mindfulness. Body scanning is associated with enhancing well-being and promoting relaxation in children [41].

In session 5, in addition to reviewing the previous techniques, a trust exercise was carried out in pairs to continue promoting the exploration of movement and mindfulness and increase trust between them. In mind–body programs, a key focus is on developing self-confidence [41].

In session 6, they were introduced to emotions and the difference between who we are and how we find ourselves. They performed a guided meditation, imagining walking through a forest, and were taught the lumberjack asana. This yoga posture involves standing and simulating the motion of chopping wood, which is designed to release tension and express emotions. Guided meditation aims to achieve positive physiological effects while also fostering an understanding of emotions and how to express them appropriately, which supports both physical and emotional self-regulation [43].

In session 7, they continued with the work on emotions, were taught the murti mudra to reduce fatigue, and again performed a body scan, but this time, it was slightly longer than in previous sessions. Mudras stimulate the endorphin system, leading to pain reduction, stress relief, and improved concentration [44].

In session 8, the work on emotions continued, and an exercise consisting of observing thoughts was carried out to continue promoting mindfulness, identifying cognitions and emotions, and enhancing each child’s self-knowledge of the inner world. In addition, guided visualization exercises were performed with the aim of reducing anxiety by influencing the autonomic nervous system [32].

### 2.4. Compilation of Information

Data were collected between 13 and 16 December 2021, through 7 face-to-face focus groups (FGs) with the children and face-to-face semi-structured interviews with the teachers. The focus groups were composed of between 6 and 9 children (four focus groups in one classroom and three focus groups in another classroom). In order to participate, the Center Management (on behalf of the child’s parents or legal guardian) and the teachers had to read the information sheet and sign the informed consent so that the confidentiality and anonymity of all participants were guaranteed. The mean duration of the interviews was between 10 and 15 min, and the mean duration of the FGs was between 12 and 15 min. The interviews were conducted by a physical therapist with expertise in child physiotherapy, extensive experience in qualitative research, and more than 25 years of experience in mind–body techniques. She ensured continuous contact and listened attentively and with interest in the participants’ contributions. She flexibly followed a script prepared in advance (Appendix A and Appendix B). The interviews with the FGs were conducted in the students’ classrooms. To facilitate a climate of trust, they were conducted in the presence, but at the appropriate distance, of their teachers and the physical therapist who had guided the sessions. Before each interview, the objective of the interview was explained to the participants, and the fact that it would be recorded to facilitate data collection and subsequent analysis. The interviews were audio-recorded and transcribed word for word. They were conducted until data saturation was achieved, i.e., when no new themes were identified and the data began to be repeated, indicating an adequate sample size was reached.

### 2.5. Data Analysis

The data analysis was performed with the help of ATLAS.ti 22 software. The phases described by Braun and Clarke in 2020 [45] were followed. Phase 1: Familiarization with the data, consisting of a complete reading of the transcripts and annotations to obtain a general sense of the data. Phase 2: Systematic data coding; the transcripts were coded using ATLAS.ti 22, and 51 codes were generated. Phase 3: Generate initial themes from the collected data; all codes were grouped with a common meaning. Phase 4: Reviewing the themes, ensuring they were consistent with the codes they grouped, and concept maps were generated with the help of ATLAS.ti 22. Phase 5: Define and name themes. Phase 6: Report writing, during which the most revealing examples of quotations and illustrations were selected, and the report was written relating the analysis carried out to the research question and the existing literature.

### 2.6. Rigor

The rigor of the research was ensured by following the criteria of Lincoln and Guba in 1985 [46]. Credibility: The data collection was described in detail and interpreted with data triangulation between researchers. Transferability: A detailed description of the participants’ experience, environment, and the context of data collection was made. Dependability: The methodology was described in detail. Confirmability: Fragments of the participants’ narratives were used verbatim.

### 2.7. Ethical Considerations

All participants were aware of the study’s objective, voluntary participation, and the possibility of leaving the study at any time without consequences. Data collection was carried out during school activities at the time indicated by the teachers as most appropriate so as not to influence the children’s routines. Permission was obtained from the center management, who informed the family members through the information sheet, and informed consent was obtained from everyone before data collection began. Anonymity was guaranteed through the use of codes during data collection and analysis. Custody and processing of personal data was ensured per current European regulations and the Personal Data Protection Act.

## 3. Results

After analyzing the transcripts of the focus groups (FGs) and interviews, 51 codes were established. Three main themes emerged that describe, from the perspective of the children and teachers, the needs detected to embark on a physical–emotional physiotherapy program, the benefits detected, and the motivations for the program to continue in the future: Theme 1: Competitiveness and uneasiness in children: it is necessary to do something; Theme 2: The seeds sown and the fruits harvested; Theme 3. It is necessary to move from the eventual to the structural (Table 2).

### 3.1. Theme 1: Competitiveness and Restlessness in Children: It Is Necessary to Do Something

Both teachers stated that their group of students presented particular difficulties, being labeled as “complicated”, “restless”, “very competitive”, and “talkative” at the faculty meeting the previous year. The need for a physical–emotional physiotherapy intervention was detected:

*“…the first-year classes last year were tricky and, well, it did occur to us, in an evaluation session, that maybe F* [the physical therapist who planned and implemented the program] *could come to our classes too, because we saw results in other classes”.*

(TEACHER_1)

The teacher participants indicated that they had observed a significant lack of attention and general concentration on the part of the children, as well as emotional management problems and great competitiveness among them. They said they knew about the mind–body program through references from colleagues who had implemented it in other classes. This made them think that their students could also benefit from it:

*“…it’s a really competitive group and, well, they wanna finish super quick to be the first…”.*

(TEACHER_1)

*“Look, they’ve got, generally speaking, a huge lack of attention. So, they also started doing this* [referring to the program] *in other courses last year”.*

(TEACHER_2)

The participating teachers stressed the importance of conducting this program now more than ever, in the aftermath of the pandemic, as the situation caused by COVID-19 had taken its toll on many children. They found that children had become more fearful and that the development of both physical and social skills had been affected:

*“…it caught them when they were in kindergarten, at five years old, you know, a huge time for play and making friends”.*

(TEACHER_2)

### 3.2. Theme 2: Seeds Sown and the Fruits Harvested

This topic collected the experiences of the participants and the teachers after participating in the program sessions. The benefits felt by the children in the first person and those referred by the participating teachers in different areas of the children’s development, not only at an academic level but also at an individual level, were recounted.

#### 3.2.1. Subtopic 1: Expression and Socialization Are Essential in the Academic Environment

The teachers emphasized the value of emotional expression in general, but specifically in this age group. They pointed out how important it was for them to learn to identify what they felt and how to express it, and that this was true for their students owing to the program:

*“…when they’re in a stressful situation that gets them mad, sad, or something that scares them, they can spot which part of their body might be tensing up and even get a handle on that body stuff”.*

(TEACHER_1)

Around the age of 7, self-regulation takes on special importance. There are significant physical and mental changes at this age, and children also begin to have knowledge tests. Moreover, these are often accompanied by stress and even anxiety. In addition, they are at a critical time for socialization among themselves. When faced with stressful or anxious situations, children feel that they have been able to self-regulate by applying the tools they have learned:

*“Well, I really liked the anger thing, how to get rid of it, ‘cause I was always super mad, and I’d start yelling, and I loved learning how to control it.”.*

(P11_2A)

*“And, after doing the lumberjack, how do you say you feel?”.*

(MODERATOR)

*“Happy…Quiet”.*

(P7_2A)

*“And why do you do that one* [when the child explains the lumberjack asana]*?”.*

(MODERATOR)

*“Because sometimes I am angry, very angry”.*

(P21_2B)

*“Very angry…”.*

(MODERATOR)

*“And I have to take it off, the anger”.*

(P21_2B)

*“That’s good, that’s good. And, when you get the anger out, how do you feel?”.*

(MODERATOR)

*“Pretty good”.*

(P21_2B)

Both teachers and students reported that the work on mindfulness and body awareness resulted in children being more focused and finding it easier to concentrate:

*“For example, when doing homework, they can focus more; they used to chatter a lot. They can sit better and have a better posture. Like, in sorting out arguments and stuff, they already talk about it differently, and at some point, you gotta say, “F* [referring to the physical therapist] *comes with this and that…”* [referring to the fact that she reminds them of what they’ve learned with the physical therapist, and it’s helped them to chill out]”.

(TEACHER_1)

*“…they’re more dialed in…”.*

(TEACHER_2)

*“…I concentrate better”.*

(P16_2B)

The mudras were also great drivers of a sense of greater focus on the present, which, coupled with conscious and controlled breathing, meant that they were able to be more concentrated, attentive, and centered on the task they were doing in the present:

*“…they can concentrate more, it’s just that they used to talk a lot…”.*

(TEACHER_1)

*“Mudra, is it called? Okay… and that, what is it for? What is it for you?.*

(MODERATOR)

*“To concentrate”.*

(P19_2A)

#### 3.2.2. Subtopic 2: Benefits Go Beyond Academics

The students learned to recognize various possible emotions they may feel. These tools are aimed at improving the children’s state of activation, seeking relaxation, stillness, and tranquility, and improving their rest. The mindfulness work helped them to become aware of how their body felt in the present moment and how they felt emotionally. They could associate an emotion or several emotions with the sensations in their body. As a result, they improved their ability to self-regulate. They began to be aware of what emotion they were feeling and where and how to apply the necessary techniques to change it. The strategies that allowed the children to express their feelings were the ones they liked the most. They highlighted the lumberjack asana, which consists of a yoga posture that favors the reduction of muscle tension and stress, producing well-being:

*“I did the lumberjack thing at home* [referring to the asana]*; I did it ‘cause I felt kinda mad at my brother and (…) I chilled out”.*

(P3_2A)

*“When my sister hits me or tells me ugly things of swear words, I get angry, and I do it with her* [lumberjack asana] *and (…) then it gives me joy”.*

(P4_2A)

Other children did not refer to a specific anger but alluded to a constant attitude, such as always having much anger. The asana had taught them to manage that feeling:

*“Well, I really liked what anger was, how to get rid of it, because I was always very angry and I would start screaming, and I loved learning to control it”.*

(P11_2A)

*“And, after doing the lumberjack one, how do you say you feel?.*

(MODERATOR)

*“Then I feel happy… calm”.*

(P7_2A)

At this age, socialization and play are fundamental, especially through relationships with their peers. They especially liked group or paired techniques.

*“I like it when we went down, and one went with his eyes closed, and the other with his eyes open, and one guided the other, and the other guided the one”.*

(P16_2B)

*“I also like it a lot because it calms me down, and I have a lot of fun playing lumberjack with my friends”.*

(P4_2B)

Both teachers explained that they now saw that the children were calmer and more relaxed and observed that, if this was not the case, they put into practice some of the techniques they had learned to relax. The students also reported this and pointed out that this stillness helped them continue their daily activities, face an exam, or concentrate more. Meditation and conscious breathing stood out among all the techniques used to achieve a feeling of stillness and relaxation:

*“Ahh, you like to keep your fingers together and breathe. And why do you like that?”.*

(MODERATOR)

*“It makes me feel better (…), I feel it calms me down (…), my body relaxes”.*

(P12_2B)

*“Breathing… and what do you do the breathing thing for?”.*

(MODERATOR)

*“To put me at ease when I am nervous”.*

(P16_2A)

According to some children, the guided imagery also contributed to this stillness. It allowed them to develop their imagination of other places outside the classroom, to feel that they could explore other spaces, feel other emotions, and then share them in a group or at home:

*“So, what was the one in the forest? Why did you like it? Let’s see, tell us about it”.*

(MODERATOR)

*“Because it was about nature, and I like nature (…). F* [the physical therapist] *told us to imagine things with that or other things, and that was it (…). I felt calm”.*

(P20_2B)

The students emphasized that they felt joy and fun owing to the novelty of the program, the possibility of socializing with peers, and learning new techniques, and that everything was combined as a game with imagination:

*“I loved it (…) because it was a lot of fun, and F* [the physical therapist] *had played a lot of fun games for us”.*

(P18_2B)

*“It gave me a lot of laughter.”* [the lumberjack asana].

(P14_2A)

*“Did it make you laugh? But you* [seeing the girl’s expression, she probes], *do you normally laugh, or don’t you?”.*

(MODERATOR)

*“No”.*

(P14_2A)

*“Don’t you usually laugh? And so, it made you laugh?”.*

(MODERATOR)

*“Yes”.*

(P14_2A)

Realizing what they feel and being able to express it has more benefits than the individual children experienced, such as the much-desired improvements in empathy among them, as the teachers mentioned:

*“…they have also improved empathy between them, and I am delighted…”.*

(TEACHER_1)

### 3.3. Theme 3: It Is Necessary to Move from the Eventual to the Structural

All the changes experienced by the children and their teachers were positive. Furthermore, it is customary to express the need and the hope that these changes can occur in other children of future generations and these same children for the following courses.

#### 3.3.1. Subtopic 1: Children Transfer What They Learn to Their Homes

According to the participants outside the sessions, one of the most practiced techniques was the lumberjack asana, with which they worked on emotional expression, specifically, the expression of muscular and emotional tension and anger, and thus, they said, achieving a sense of calm and tranquility. They also practiced mudras to reinforce the ability to concentrate, meditation, and breathing techniques. The teachers pointed out that the children were applying the techniques at home:

*“… and I know that it is, I know that in some houses it is being done…”.*

(TEACHER_2)

Many students explained that they had practiced some of the techniques they had learned at home and on their initiative. Some mentioned that they only practiced them when feeling nervous, while others taught the techniques to their parents so they could practice together. Additionally, some did them with their siblings, who were already familiar with the techniques because the program had been conducted with older children in a pilot test the previous year.

*“And do you guys ever do it at home?”.*

(MODERATOR)

*“Always, because after lunch… As my mother, one year I was so good that for my birthday I got a machine… And then, as I downloaded YouTube… Always, after eating, when I do this, I put on relaxing music… And I concentrate better* [referring to the meditation done in class]*”.*

(P16_2B)

Some children engaged in visualization exercises combined with conscious breathing, integrating mindfulness practices with body awareness. Several participants noted that they performed these exercises independently at home, without the physical therapist’s presence. This adds a unique dimension to the intervention, as it not only has an immediate impact when conducted at school but also empowers children to manage their emotions in various situations:

*“… that practice* [breathing]… *you imagine you are in your forest* [guided imagery] *… do you ever do it by yourself?”.*

(MODERATOR)

*“Yes. (…) I even draw pictures of it!”.*

(P15_2A)

#### 3.3.2. Subtopic 2: The Desire to Consolidate These Actions in the School Center

Given the experience and the positive changes, both teachers expressed complete confidence in the program. They felt committed to trying to maintain the benefits for the participants and had transferred some of the learning to their classes by reinforcing what the children had learned in the program:

*“…in class, we do, every day, in the middle of the morning, before recess, we take a break, and then we do drink water, hydrate ourselves, become aware of the body, do some breathing, do mudra frequently…”.*

(TEACHER_1)

*“…after the control *[exam]*, to change things up a bit and break up, since today we had a language control, we did the pebble exercise [exercise to encourage the expression of emotions] …”.*

(TEACHER_2)

Both teachers expressed their desire to include it in the teaching curriculum. They confessed the difficulties they had in devoting time to it at present, despite knowing the benefits, due to the large amount of mandatory daily tasks and the much content to be taught:

*“Let’s see, there… I would like to, maybe sometimes, take it up again more, but well, time…”. “Yes, we are very tight with the curriculum… so, yes, I was telling you that this should be included in a transversal way… but you cannot always dedicate the time to it…”.*

(TEACHER_1)

As an essential and definitive step forward for its official implementation in the students’ curriculum, the teachers emphasized how important it was that they trusted in the program. They emphasized that it was essential for them to be present in all the sessions and to practice each of the proposals made by the physical therapist. In this way, they perceived the program’s benefits in their flesh, thus feeling motivated to dedicate time to this technique in class. They said it would also be highly recommended that the physical therapist train the teachers. In this way, it would be possible to try to give continuity and facilitate the consolidation of the benefits for the children of everything they learn:

*“Of course, yes, because, of course, at the end of the day, either you feel it and see that it is something good, and it is something that can be done and that can be integrated in a transversal way, or if you don’t understand it, you will say “I don’t want to add more things, we already have enough” … In the end, it depends a little on the teacher, on the experience they have, and how they live it… How he/she lives all this and the importance he/she gives to it”.*

(TEACHER_1)

*“And it is perfect that in parallel to this, F* [the physical therapist] *is also training us teachers, so I think it is an action, and the good thing is that it is a very global intervention and that it is not isolated and so on… Yes, so I think it is important. I also think it is important that we can introduce it, as I said, in a transversal way in the curriculum, that teachers are given the tools to be able to do this”.*

(TEACHER_2)

## 4. Discussion

The present study aimed to explore the perceptions of 6- to 7-year-old children and their teachers who participated in a program led by the school’s physical therapist that focused on mindfulness, body awareness, conscious breathing, and emotional management.

Three themes were generated from the analysis of the data. The first theme reflected the needs detected in children of this age group in general and, specifically, in these two groups of students who were labeled as “complicated”, “restless”, “talkative”, and “very competitive”. In recent years, it has been observed that children face constant overstimulation. As early as 2013, it was observed that this generation suffered the most stress compared to previous generations and also developed fewer coping skills [47]. The study revealed that teachers reported students exhibiting a lack of attention and concentration, with notable restlessness and nervousness at the beginning of the course. They also reported that the faculty discussed this at the course preparation meeting. The pandemic had caused physical and psychological health problems in children and adolescents [48], as reported by the teachers in the interviews. They also indicated that they perceived that children had become more fearful, and many of them had had their social skills affected. 

The second theme showed the benefits reported by both children and teachers after participating in the workshop in which they learned a wide range of tools: mindfulness in body awareness and conscious breathing, mudras, asanas, and guided visualizations. Filipe et al. [49], in their systematic review, analyzed different interventions based on various mindfulness techniques in order to identify which of them had more benefits. They concluded that some of these involved improvements in attention, stress regulation, and understanding of the emotional state in the present moment. They also determined that body scanning, one of the techniques with which body awareness is developed, was associated with the promotion of well-being and a decrease in anxiety. The study found that children reported experiencing a state of generalized relaxation from the body scanning technique. The study showed that both children and teachers reported enhanced concentration and attention resulting from body awareness and mindfulness techniques. It is crucial to improve the understanding of one’s current emotional state at this age. This understanding and recognizing how emotions affect the body physically (such as causing tension in specific areas) is fundamental. It serves as the foundation for emotional expression. In addition, children have learned that this emotion is transitory and does not define them. For example, if there is a moment when they are nervous or uneasy, such as before an exam, they know how they are at that moment if their body muscles are tense or they breathe faster. However, they understand this is temporary; they are not inherently nervous children but instead experiencing nervousness. They also recognize that they can apply some techniques they have learned to accept or express the feeling and allow it to pass. Dunning et al. [50], in their meta-analysis, assessed the efficacy of using mindfulness-based intervention to improve mental health and well-being in young people. They concluded that mindfulness-based interventions greatly moderated negative behaviors in children (such as talking when they should not or becoming aggressive), decreased stress and anxiety, and increased relaxation and stillness. As a more global result, the teachers observed a significant transformation in the classroom dynamics. They explained that, after several sessions, the students had undergone a notable shift from “being a restless and restless and unfocused class” to becoming more attentive with better behavior on a regular basis. Furthermore, they reported that these benefits were noticeable not only in the students’ academic performance but also in their behavior among peers, with some students even showing greater empathy.

Also, the study participants reported benefits from breathing-based exercises. The review paper on yoga and breathing exercises by Das et al. [51] analyzes the effect of slow and rhythmic breathing in yoga in children with asthma, concluding that they have beneficial effects. These results are similar to those obtained previously in a systematic review by Jayawardena et al. [52]. On the other hand, Thomas et al. [53] found that the breathing techniques used in yoga and relaxation techniques positively affect stress levels, physical activity, and behavior in school-aged children. In addition, at the physiological level, Khalsa et al. [54] showed that slow, rhythmic breathing promoted the release of prolactin and oxytocin, hormones that can promote feelings of friendliness, calmness, and bonding with others. Breathing is included in most mindfulness-based interventions, such as yoga or meditation. Chui et al. [55] demonstrated that in this practice, the body’s breathing pattern is often slowed down, resulting in an intensification of vagus nerve action that reduces negative emotions and regulation of the body to stress. The present work demonstrates these findings through reports from children and guardians, including improvements in socialization and feelings of calmness, happiness, tranquility, or joy. Slow and rhythmic breathing in the form of exercises adapted to the children, like breathing with colored stones, could even improve the children’s ventilatory activity. However, this was not asked in the interviews.

Taliaz et al. [56] showed that meditation increased the thickness of the left hippocampus, the brain region responsible for emotional regulation and cognition and pivotal in resistance to chronic stress. Furthermore, after a thorough review of the literature on the subject, Stephens et al. [57] determined that mindfulness meditation, in the long term, involves a decrease in the activity and size of brain areas related to anxiety, such as the amygdala, and an increase in the prefrontal cortex and insular cortex, both of which are controllers of emotional management. The study results indicate that participants reported better emotional regulation and management in children as well as a decrease in stress and anxiety.

Dunning et al. [50] explain in their meta-analysis that children develop self-regulation significantly at this stage, so its practice and training are very beneficial. Research, such as that of Janz et al. [58], shows that self-regulation is related to developing empathy and conscientiousness, general social and emotional well-being, and academic success. Furthermore, low levels of self-regulation in young children predict negative behaviors, elevated stress levels, and school failure. A review by Bockmann [59], which examines the effects of mindfulness-based interventions on self-regulation in early childhood, shows that applying such interventions supports them in the development of self-regulation. Therefore, our results agree with the findings of these studies since both teachers and children reported improvements in their self-regulation. This can be seen, for example, when some of the children said that they applied one of the techniques (both at school and home) when they were angry and then calmed down again. In addition, one of the teachers related this improvement in self-regulation skills to the improvement in empathy she had observed in some of the children, which is a remarkable boost to development during this stage of life, as alluded to by Sappok et al. [15].

The program conducted by the school’s physical therapist includes several techniques that can be included in those coming from yoga, such as breathing and asanas. There was a qualitative study on how the practice of yoga influenced young people in an improvement in emotional regulation and expression, how breathing contributed positively to their lives, and how it allowed them to reduce their anxiety and feel calmer and happier [60]. Emotions motivate and guide our actions, and being aware of them is fundamental for their self-regulation and expression. The study observed improvements in children’s capacity to understand the current state and its effects on their bodies as a result of the program. On the one hand, the review by Stephens et al. [57] establishes that children who practice yoga have a greater sense of self-awareness and self-confidence, improved concentration, attention, self-esteem, empowerment, and good mental health, all of which were reported by our participants. On the other hand, asanas are specific yoga body postures that are usually taught along with some breathing or meditation techniques. In their review, they explain that asana practice, along with slow and rhythmic breathing, promotes parasympathetic and vagal tone [57]. This entails decreased heart and respiratory rates and cortisol levels, among others, and is related to increased relaxation and well-being. The research findings, based on student reports, indicate that asanas, specifically the lumberjack pose, help reduce both muscular and mental tension, decrease stress, and generate a sense of well-being and joy in some cases.

On the other hand, Hoag et al. [61] conducted a randomized controlled trial comparing the effectiveness of guided imagery vs. virtual reality on pain and anxiety before and after undergoing painful and distressing procedures. They found that guided imagery was associated with a decrease in anxiety and subsequent relaxation. Weydert et al. [62] showed that this technique is especially effective in children of these ages, given their capacity for creative and active imagination and high suggestibility. These findings remind us of the children in our study who said they experienced calmness when practicing guided imagery and visualization techniques.

This type of intervention also leads to an improvement in postural control, as Orendorz-Fraczkowska et al. [63], corresponding to what happens in the study, stated that children improved their postural hygiene owing to the techniques, they were aware of how their bodies were and could modify it, for example, as the teachers reported, by sitting better and with a better posture. According to Nijhof et al. [64], play is essential in children for proper physical, social, emotional, and cognitive development. As Yogman et al. [65] reported, it allows them to relate to peers by developing social and emotional skills and generating feelings of euphoria, joy, and fun in them, producing endorphin release and having multiple benefits at a physical level. Combining the different mind–body techniques with play at these ages is fundamental since, apart from the benefits of the intervention itself, benefits related to the game itself arise, as in the present study, the children expressed fun and joy through the interviews they were given.

As discussed, multiple studies have shown the benefits of yoga, mindfulness, and mindfulness programs targeting this age group in isolation. Carsley et al. [66] concluded in a meta-analysis that mindfulness interventions have a more significant effect when combined. This study represents a step forward by providing a platform for participating children and their guardians to express the perceived positive impact on their lives through a mind–body program. Additionally, the study is notable for its use of a program combining several mind–body interventions.

The last theme generated in our study was a declaration of intentions and commitments by the teachers participating in the program. They wanted this workshop to last longer and to be able to reach more children and teachers since the benefits were not only perceived in the school environment but also outside it. Although aware of the difficulties involved, the teachers expressed the desire to implement it regularly and regulate it in the school curriculum several times. As emphasized in other studies [57,67], the teachers noted that giving continuity to the children’s work outside the workshop is essential. To achieve lasting benefits with this type of intervention, combining them and transferring them to all the children’s environments where they can use, practice, and train them is critical.

### 4.1. Limitations of the Study

The results of this study cannot be generalized to all children of this age group, or to all programs related to mindfulness, body awareness, and emotional management. This limitation in generalizability stems from several factors. Individual differences among children, including varied emotional and cognitive profiles, diverse cultural backgrounds, and unique environmental contexts, may significantly influence responses to these interventions. Additionally, potential variations in the quality of implementation and the lack of long-term evaluation further complicate generalizability.

However, the present study does provide valuable insights into how the program has affected the participants and the benefits it has brought them. It contributes to the understanding of the potential benefits of these techniques, which may facilitate adaptations for wider application. By adjusting the content and approach to the specific needs of other groups of children, and by training those working with children, mindfulness practices can be integrated into daily routines. The basic principles of these mind–body interventions can be effectively adapted to meet the diverse needs of various student populations, thus maximizing the potential benefits for children beyond the original sample.

This study focused on children and the school environment. Future research could benefit from examining the opinions and experiences of family members, which would be valuable to learn more about the program’s reach beyond the school environment. Families play a pivotal role in reinforcing the skills and strategies learned in school, and their perspectives could shed light on how these interventions are applied in home contexts. Understanding familial dynamics could help identify potential barriers or facilitators to the successful implementation of the program, offering a more comprehensive view of its effectiveness. Additionally, family involvement may enhance children’s emotional and behavioral outcomes, as supportive home environments are known to contribute to overall well-being. By integrating the experiences of families, researchers could evaluate the program’s reach and sustainability beyond the school setting, ultimately leading to more robust and effective interventions that support children.

### 4.2. Potential Clinical Implications

The findings from this study suggest several potential clinical implications regarding the incorporation of mindfulness and body awareness techniques into early childhood education. These implications span various aspects of child development and well-being, from mental health to academic performance.

In terms of mental and emotional health, the study indicates that implementing these techniques at an early age could be particularly beneficial. The positive outcomes observed in 6- to 7-year-old children suggest that early intervention might potentially prevent the development of more severe emotional and behavioral issues later in life. The study demonstrates the potential of these techniques in fostering emotional intelligence and self-regulation skills, which are particularly crucial at this developmental stage. These skills could have long-lasting effects on children’s mental health and social interactions. Moreover, the observed reduction in stress and anxiety levels among participants suggests that these techniques could serve as effective coping mechanisms for children facing academic and social pressures, potentially contributing to long-term mental health resilience.

From an academic perspective, the reported improvements in concentration, attention span, and overall behavior suggest that these programs could positively influence academic performance. This implies that mindfulness techniques could be integrated into educational strategies to support learning outcomes. The improvement in interpersonal relationships and empathy reported by teachers also points to the potential of these programs in fostering better peer relationships and social competence, which are crucial for long-term psychological well-being and academic success.

The study also hints at potential physical health benefits. While not directly measured, the potential improvements in postural control and body awareness suggest possible benefits for children’s physical health and development. This underscores the holistic nature of the approach, which combines mindfulness, body awareness, conscious breathing, and emotional management techniques to address children’s developmental needs comprehensively.

The positive reception from teachers implies that training educators in these techniques could enhance the overall classroom environment and teacher–student relationships. Furthermore, the reported practice of techniques at home indicates the potential for these interventions to bridge the gap between school and home environments, potentially amplifying their effects.

It is important to note that while these findings are promising, they warrant further exploration and research. Future studies should focus on long-term outcomes, optimal implementation strategies, and the potential for these techniques to address specific developmental challenges in early childhood. Additionally, investigating the impact of such programs on children with diverse needs or from various socio-economic backgrounds could provide valuable insights for tailoring interventions to different populations.

In conclusion, this study suggests that mindfulness practices in educational settings may have the potential to facilitate holistic development and support children’s mental health. However, more research is needed to fully understand and validate these potential benefits. The multi-faceted nature of the intervention strategy used in this study may offer advantages over single-technique approaches, but comparative studies would be necessary to confirm this hypothesis.

## 5. Conclusions

This study analyzed the impact of a combined program of mind–body interventions led by a physical therapist on second-grade primary school children and their teachers. The research identified specific challenges faced by the children, including difficulties with attention and concentration, heightened competitiveness, and a need for improved socialization. These observations helped tailor the program to address these particular needs.

The findings revealed significant positive outcomes from the intervention. Participants reported improvements in several key areas: self-regulation (children demonstrated enhanced ability to manage their emotions and behaviors); relaxation (the program facilitated better stress management and overall calmness); attention span (both children and teachers noted increased focus and concentration); stress reduction (participants experienced decreased anxiety and tension).

Furthermore, the program yielded benefits beyond individual well-being. It enhanced interpersonal relationships and social skills among the children, fostering a more positive classroom environment. Notably, the intervention generated feelings of joy and enjoyment, indicating its potential to make learning and personal development more engaging and fun for these students.

In summary, the study results suggest that the mind–body intervention program significantly contributed to the children’s emotional well-being and social skill development. These findings underscore the value of integrating such holistic approaches within the school setting. By addressing both cognitive and emotional aspects of child development, this type of intervention shows promise in creating a more supportive and effective learning environment. Future research and educational policies should consider the potential long-term benefits of incorporating similar programs into standard school curricula. The observed improvements in self-regulation, stress management, and social skills have broader implications for children’s overall health and well-being. Enhanced emotional regulation and stress reduction may contribute to better mental health outcomes, potentially reducing the risk of future psychological issues. Improved social skills and classroom dynamics could lead to a more positive school experience, potentially impacting academic performance and long-term educational outcomes.

Furthermore, the benefits of these interventions may extend beyond the individual child to impact family dynamics and broader social contexts. As children learn to better manage their emotions and stress, this could lead to more harmonious home environments and improved family relationships. On a societal level, early interventions that promote emotional well-being and social skills may contribute to creating more resilient and adaptable future generations, potentially reducing the burden on healthcare systems and improving overall community health.

Future research should focus on long-term outcomes, optimal implementation strategies, and the potential for these techniques to address specific developmental challenges in early childhood. Additionally, investigating the impact of such programs on children with diverse needs or from various socio-economic backgrounds could provide valuable insights for tailoring interventions to different populations, further enhancing the potential health benefits across diverse communities.

## Figures and Tables

**Table 1:** Characteristics of the participants of the focus group.

Participant	Sex	Age
P1_2A	Woman	6
P2_2A	Man	7
P3_2A	Man	6
P4_2A	Woman	6
P5_2A	Man	7
P6_2A	Man	6
P7_2A	Man	7
P8_2A	Woman	7
P9_2A	Woman	6
P10_2A	Man	6
P11_2A	Woman	6
P12_2A	Woman	6
P13_2A	Man	6
P14_2A	Woman	7
P15_2A	Man	7
P16_2A	Woman	7
P17_2A	Woman	6
P18_2A	Man	6
P19_2A	Man	7
P20_2A	Woman	6
P21_2A	Man	7
P22_2A	Man	7
P23_2A	Woman	6
P24_2A	Man	6
P25_2A	Woman	6
P1_2B	Man	7
P2_2B	Man	7
P3_2B	Woman	6
P4_2B	Woman	6
P5_2B	Woman	6
P6_2B	Woman	7
P7_2B	Man	6
P8_2B	Man	7
P9_2B	Man	6
P10_2B	Man	6
P11_2B	Man	6
P12_2B	Man	7
P13_2B	Woman	6
P14_2B	Woman	7
P15_2B	Woman	7
P16_2B	Man	7
P17_2B	Man	6
P18_2B	Woman	6
P19_2B	Woman	7
P20_2B	Man	7
P21_2B	Man	6
P22_2B	Woman	6
P23_2B	Woman	6
P24_2B	Woman	7
P25_2B	Man	6
P26_2B	Man	6

Source: Prepared by the authors.

**Table 2:** Topics and subtopics.

Subject	Subtopic
1. Competitiveness and restlessness in children: Something needs to be done	
2. Seeds sown and fruits harvested	2.1. Expression and socialization are essential in the academic environment
	2.2. Benefits go beyond academics
3. It is necessary to move from the eventual to the structural	3.1. Children transfer what they learn to their homes
	3.2. The desire to consolidate these actions in the school center

Source: Prepared by the authors.

## Data Availability

The data presented in this study are available on request from the corresponding author. The data are not publicly available due to ethical reasons.

## Appendix A

Teacher’s Interview Questions Script.

**Interview Stage**	**Target**	**Content and Sample Questions**
Introduction	My intention	These interviews aim to learn about the workshops’ impact on the children.
	Information and ethical aspects	The conversation needs to be recorded in order to analyze the data. Consent is requested to ask the questions and record the interview.
	Consent	Sign the informed consent form.
Start	Introductory question	You have already attended eight sessions of the Mindfulness Program; what is your general feeling about the workshops?
Development	Conversation Guide	You will be asked about the techniques/tools the physical therapist has introduced in class and which have been most helpful to the children. She will be asked about her feelings about what the children have learned and about the benefits she perceived as reported by the children, the physical therapist, and the family.
Closing	Last question	You are allowed to add something if you wish.
	Acknowledgments	Thank you for your participation

## Appendix B

Children’s Question Script.

**Interview Stage**	**Target**	**Content and Sample Questions**
Introduction	Our intention	The purpose of these interviews is to deeply understand how the children are experiencing the workshops, what they are learning, and whether it is helping them in their daily lives.
	Information and ethical aspects	The conversation needs to be recorded to analyze the data. The children are informed that they are being recorded. In addition, by delegation to the Center Management, the parents have previously signed an informed consent form.
	Consent	Sign the informed consent form.
Start	Introductory question	What is the technique you have learned that you like the most?
Development	Conversation Guide	Why do you like that/those? When do you do it? Do you practice it with anyone? Do you do any in class when the physio is not there?
Closing	Last question	They are allowed to add something if they wish to do so.
	Acknowledgments	Thank you for your participation.
